# Supplementary material for: A quick and robust method for quantification of the hypersensitive response in plants
Source: PeerJ. 2015 Dec 1;3:e1469. doi: 10.7717/peerj.1469 (PMC4699783; doi:10.7717/peerj.1469)
Supplement: Supplemental Information 1 — The document describes a step by step protocol for the method described in the article. [file peerj-03-1469-s004.pdf]

Vacuum infiltration of *Pseudomonas syringae* into leaf discs

Based on Johansson and Nilsson *et al*. 2015 “A quick and robust method for quantification of the hypersensitive response in plants”.

Materials: Plastic 50 ml centrifuge tubes
10 mM $MgCl_2$
Plastic bacterial inoculation loops
Petri dishes
6 well cell cultivation plates
Tea strainer
Vacuum concentrator (e.g SpeedVac) connected to a vapor trap
Conductivity meter

Before start

- Exactly 24 hour prior to experiment start: Restreak a loopfull of *P. syringae* on KB plates with appropriate antibiotics. Grow in room temperature over night.
- Day before or just before experiment start: Rinse one 6 well culture plate for each plant line/treatment to be investigated three times in deionized water. Culture plates can be reused several times.
- Add 10 ml of ddH_20 to each plate well and measure conductivity.

Preparation of leaf discs and bacterial suspension

- Make leaf discs from 4-6 weeks old Arabidopsis plants using a cork borer by punching them out against a piece of Styrofoam. If possible, avoid the midvein. Put the discs into a 50 ml plastic centrifuge tube. Approximately 35-40 discs per plant line are needed.
- Prepare the bacterial solution by suspending a loopfull of the over-night culture of *P. syringae* in 10 mM $MgCl_2$. Measure OD_{600} and dilute to appropriate concentration. OD_{600}=0.05 works well for most applications.
- Add 20 ml of the bacterial suspension to each centrifuge tube containing leaf discs. Put the lid back on the tube but only tighten it loosely to allow free exchange of air between the tube and the surrounding. Swirl the suspension to separate the discs from each other.

Vacuum infiltration

- Put the tubes with the discs into a rotorless SpeedVac vacuum concentrator. Make sure that the vacuum concentrator is connected to a vapor trapping system to avoid aerosolized vapor containing bacteria in the laboratory. Lower pressure until the bacterial suspension starts boiling and keep as such for 10 seconds (do not turn on the centrifuge). Turn off the vacuum. Note that most of the discs will be infiltrated when they are returned to atmospheric conditions. If the infiltration efficiency is low, the vacuum treatment may be repeated.
- Pour the infiltrated discs into a tea strainer and rinse under a stream of deionized water.
- Further wash the discs by transferring them into Petri dishes filled with deionized water.
- After 10 minutes of washing, transfer 4 fully infiltrated discs (sunken discs) using plastic bacterial inoculation loops to each plate well. Prepare 6 replicates per plant line.
- Measure conductivity hourly.

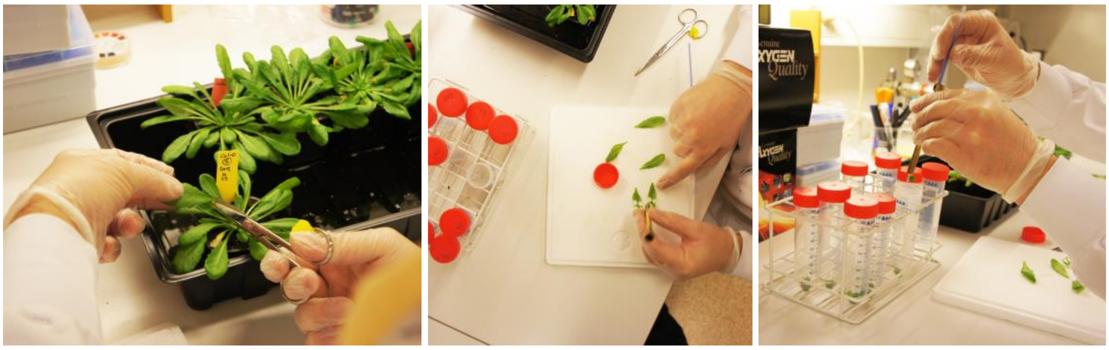

Step 1. Making leaf discs
Leaf disc from 4-6 weeks old Arabidopsis plants are cut out against a piece of Styrofoam using a cork borer. Discs are then put in 50 ml centrifuge tubes. Approximately 35-40 discs per plant line are needed.

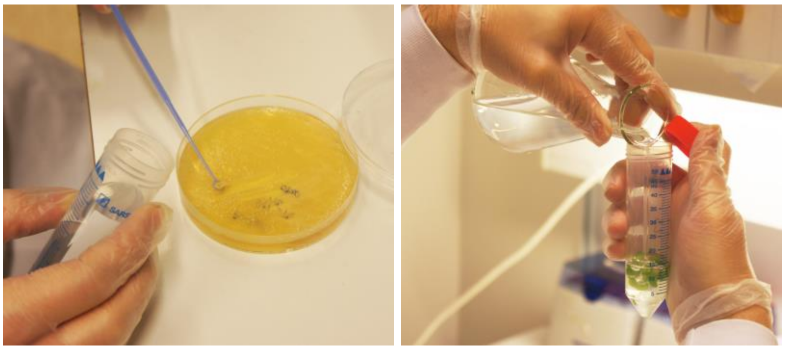

Step 2. Adding bacterial suspension
A loopfull of bacteria from a 24 hour culture of *P. syringae* is suspended in 10 mM $MgCl_2$.Optical density is measured at 600 nm and the suspension is diluted to appropriate concentration. For most application, OD_{600}=0.05 works well. Approximately 20 ml of the suspension is added to each centrifuge tube with leaf discs. The lid is closed but not completely to allow free gas exchange.

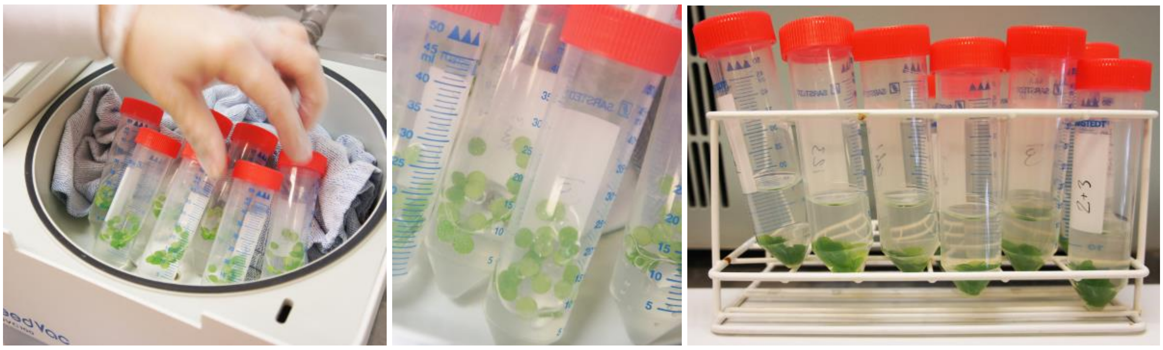

Step 3. Vacuum infiltration
Before the tubes are placed in the vacuum chamber, discs are separated from each other by swirling the tubes. Pressure is decreased until the bacterial suspension starts boiling and kept as such for 10 seconds. Discs are infiltrated when returned back to normal atmospheric conditions. Fully infiltrated discs are easily distinguishable as they lose their buoyancy.

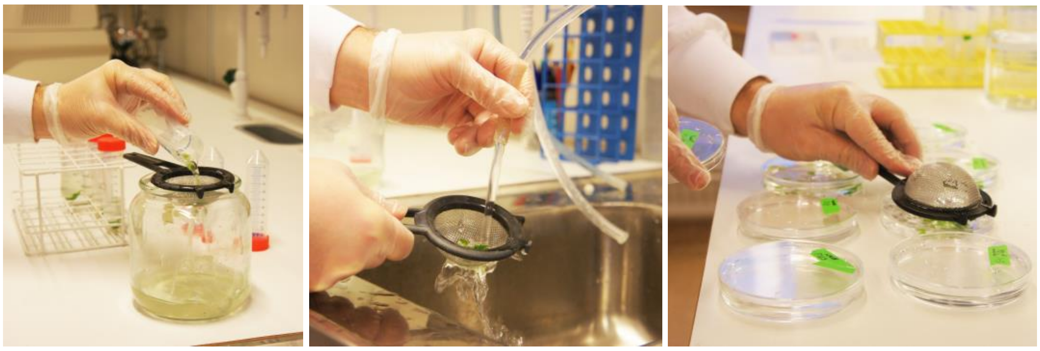

Step 4. Washing the discs

The discs are poured into a tea strainer and rinsed under a stream of deionized water. Discs are then transferred to Petri dishes containing deionized water.

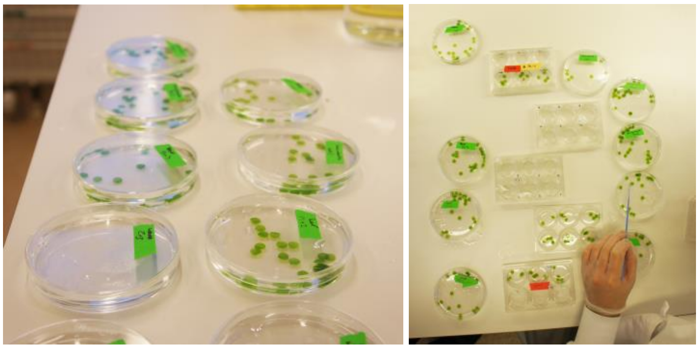

Step 5. Transferring the discs to cell culture plates

Discs are left to wash in the Petri dishes for 10 minutes. Using plastic bacteria inoculation loops, discs are moved to 6 well culture plates filled with 10 ml ddH$_2$0. 6 replicates with four discs per replicate/plate well.

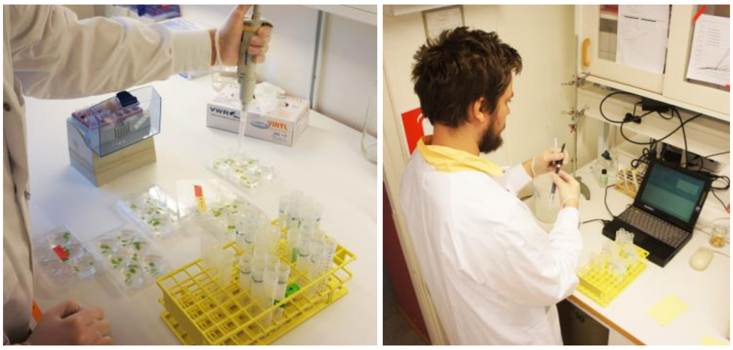

Step 6. Measuring conductivity

5 ml from each sample is transferred to 12 ml tubes and conductivity is measured every hour.
