# Supplementary material for: A quick and robust method for quantification of the hypersensitive response in plants
Source: PeerJ. 2015 Dec 1;3:e1469. doi: 10.7717/peerj.1469 (PMC4699783; doi:10.7717/peerj.1469)
Supplement: Table S1 — Nutritional composition of three different media for cultivation of Pseudomonas sp. Numbers inducate g/L media as reported by the manufacturers. [file peerj-03-1469-s005.docx]

**Supplemental table S1.** Composition of three different media used for cultivation of *Pseudomonas*. Numbers indicate g/L media as reported by the manufacturers.

|  | *Pseudomonas* agar F (King´s B medium), Biolife (Milano, Italy) | King´s B medium, Duchefa Biochemie  (Haarlem, The Netherlands) | *Psuedomonas* agar base, Biolife (Milano, Italy) |
| --- | --- | --- | --- |
| Acid digest of casein | - | - | 16 |
| Peptone | 10 | - | - |
| Peptone G | - | - | 10 |
| Tryptone | 10 | - | - |
| Proteose | - | 20 | - |
| MgCl_2_ | - | - | 1.4 |
| MgSO_4_ | 1.5 | 0.73 | - |
| K_2_HPO_4_ | 1.5 | 1.5 | - |
| K_2_SO_4_ | - | - | 10 |
| Agar | 15 | 15 | 11.5 |
