# Supplementary material for: A quick and robust method for quantification of the hypersensitive response in plants
Source: PeerJ. 2015 Dec 1;3:e1469. doi: 10.7717/peerj.1469 (PMC4699783; doi:10.7717/peerj.1469)
Supplement: Table S2 — Given in the table are the estimated parameters used for modeling electrolyte leakage from Col-0 plants inoculated with P. syringae expressing the AvrRpm1 effector. [file peerj-03-1469-s006.docx]

**Supplemental table S2. Parameter estimates from regression analysis.** Given below are the estimated parameters used for modeling electrolyte leakage from Col-0 plants inoculated with *P. syringae* expressing AvrRpm1.

| Figure reference | OD_600_ | θ_min_ | θ_max_ | θ_1_ | θ_2_ | θ_3_ |
| --- | --- | --- | --- | --- | --- | --- |
| **7 A-G** | 0.1 | 6.5291 | 102.6872 | -35.6709 | 125.7436 | -3.4899 |
|  | 0.05 | 5.9129 | 122.4777 | -33.0516 | 107.6087 | -3.2557 |
|  | 0.01 | 5.9128 | 61.6640 | -10.9686 | 16.4924 | -1.3035 |
|  | 0.005 | 5.4553 | 42.1926 | -9.1350 | 10.9128 | -0.8920 |
|  | 0.001 | 0.4727 | 22.6240 | -3.4988 | 1.4436 | 0.4727 |
| **S2 A** | 0.1 | 1.1333 | 69.0000 | -19.7635 | 50.8383 | -2.4161 |
|  | 0.01 | 0.7167 | 41.3333 | -3.4580 | 1.7204 | 0.3075 |
|  | 0.001 | 1.4000 | 15.9833 | -1.2967 | 0.2893 | 1.1395 |
| **S2 B** | 0.1 | 1.2500 | 59.3333 | -13.6431 | 26.2047 | -1.6925 |
|  | 0.01 | 0.8500 | 29.5000 | -2.6602 | 0.9125 | 0.7575 |
|  | 0.001 | 1.0667 | 11.4833 | -1.1078 | 0.1438 | 1.6599 |
